# Role of the Orexin System on the Hypothalamus-Pituitary-Thyroid Axis

**DOI:** 10.3389/fncir.2016.00066

**Published:** 2016-08-25

**Authors:** Antonietta Messina, Carolina De Fusco, Vincenzo Monda, Maria Esposito, Fiorenzo Moscatelli, Anna Valenzano, Marco Carotenuto, Emanuela Viggiano, Sergio Chieffi, Vincenzo De Luca, Giuseppe Cibelli, Marcellino Monda, Giovanni Messina

**Affiliations:** ^1^Department of Experimental Medicine, Section of Human Physiology and Unit of Dietetic and Sport Medicine, Second University of NaplesNaples, Italy; ^2^Neapolitan Brain Group (NBG), Clinic of Child and Adolescent Neuropsychiatry, Department of Mental, Physical Health and Preventive Medicine, Second University of NaplesNaples, Italy; ^3^Department of Clinical and Experimental Medicine, University of FoggiaFoggia, Italy; ^4^Department of Psychiatry, University of TorontoToronto, ON, Canada

**Keywords:** orexin, posterior lateral hypothalamic area, neuroendocrine control, HPT axis, autonomic functions

## Abstract

Hypocretin/orexin (ORX) are two hypothalamic neuropeptides discovered in 1998. Since their discovery, they have been one of the most studied neuropeptide systems because of their projecting fields innervating various brain areas. The orexinergic system is tied to sleep-wakefulness cycle, and narcolepsy is a consequence of their system hypofunction. Orexinergic system is also involved in many other autonomic functions such as feeding, thermoregulation, cardiovascular and neuroendocrine regulation. The main aim of this mini review article is to investigate the relationship between ORX and thyroid system regulation. Although knowledge about the ORX system is evolving, its putative effects on hypothalamic-pituitary-thyroid (HPT) axis still appear unclear. We analyzed some studies about ORX control of HPT axis to know better the relationship between them. The studies that were analyzed suggest Hypocretin/ORX to modulate the thyroid regulation, but the nature (excitatory or inhibitory) of this possible interaction remains actually unclear and needs to be confirmed.

## Introduction

Some recent studies in the literature have reported the existence of a novel family of biologically active neuropeptides, selectively isolated from the mammalian hypothalamus, named orexins (ORX; Sakurai et al., 1998; de Lecea et al., 1998). The ORX, including ORX-A and ORX-B (also termed as hypocretin-1 and hypocretin-2), derive from the proteolytic cleavage of a common 130-amino-acid precursor named preproorexin (Gotter et al., 2012), identified in an intracellular calcium influx assay as endogenous peptide ligands for multiple orphan G protein-coupled cell surface receptors (Sakurai et al., 1998). ORX-A was initially described as an appetite-stimulating factor following local injection of ORX-A into hypothalamic areas such as the dorsomedial hypothalamus (DMH; Dube et al., 1999; De Luca et al., 2008; Viggiano et al., 2009), and in the lateral hypothalamic area (LHA), where feeding behavior and energy balance are regulated (Russell et al., 2002; Sellayah and Sikder, 2013). de Lecea et al. (1998) and Sakurai et al. (1998) observed that ORX share structural similarity with the secretin-related peptides, so they named them hypocretin-1 and hypocretin-2 (Hcrt-1 and Hcrt-2) to identify them as a hypothalamic member of the incretin family (Gotter et al., 2012). At the same time, Sakurai et al. (1998) reported that central administration of these peptides stimulated feeding, so they renamed them ORX-A and ORX-B (from the Greek word for appetite, “orexis”). Mammalian ORX are 28 and 33-amino acid peptides, respectively, encoded by a single mRNA transcript, with a 46% amino acid sequence identity. They are encoded by a gene located in the chromosome 17q21-q24. Their actions are mediated through binding and activating of two closely related G-protein coupled receptors, respectively called orexin receptor 1 (OX1R), and orexin receptor-2 (OX2R), belonging to the rhodopsin-like family A GPCRs. These receptors display different affinity for ORX. The OXR1 has less affinity for ORX-A than for ORX-B. Conversely, OXR2 shows similar affinities for both peptides (Di Bernardo et al., 2014; Esposito et al., 2014b; Monda et al., 2014).

Studies showed that electrical stimulation of the LHA induced morphological changes in the thyroid gland (López et al., 2010). In addition, Kaufman et al. (1986) demonstrated that rats with lesions in the LHA showed lower triiodothyronine (T3) and thyroxine (T4) levels; while, Suzuki et al. (1982) demonstrated that the administration of thyroid-releasing hormone (TRH) in the LHA had a marked anorectic effect. Overall, these data suggest that ORX could modulate the hypothalamic–pituitary–thyroid (HPT) axis. This idea was supported by studies demonstrating that peripheral ORX-A administration in rats inhibits the TRH release from the hypothalamus (Mitsuma et al., 1999), resulting in a fall in the levels of thyroid-stimulating hormone (TSH; Mitsuma et al., 1999; Novak and Levine, 2009). This effect appears to be entirely mediated by the decrease in TRH levels, because both ORX-A and ORX-B failed to inhibit TSH secretion in primary cultures from rat pituitary (Samson and Taylor, 2001). It has been suggested that this lack of effect could be based on the slow metabolism of thyroid hormones (THs; Novak and Levine, 2009), which normally show a delay in their secretory responses to modulatory factors (Kim et al., 2000).

## Orexigenic System

ORX are selectively expressed by few neurons located within the lateral, dorsomedial and perifornical areas of the hypothalamus (Messina et al., 2014a; Viggiano et al., 2014). Although orexinergic peptides are produced by a discrete neuronal population with a specific anatomical origin, their projection fields have been identified in various brain areas, including thalamus, hypothalamus, cerebral cortex and brainstem (Date et al., 1999; Nambu et al., 1999; Messina et al., 2014b). Based on this unique pattern of ORX-containing fiber distribution and the functional activation of neural circuits, the pleiotropic functions of ORXs and their involvement in the coordination of multiple versatile physiological processes, such as sleep-wakefulness, arousal, energy balance, narcolepsy, glucose metabolism, gastric ulcers and thermogenesis were proposed (Szlachcic et al., 2013; Tsujino and Sakurai, 2013; Giardino and de Lecea, 2014).

Other studies suggest that the ORX system could modulate the HPT axis. In fact, the peripheral ORX-A administration in rats inhibits TRH release from the hypothalamus (Mitsuma et al., 1999), resulting in a fall of TSH levels (Russell et al., 2000). This effect appears to be entirely mediated by the decrease in TRH levels, because both ORX-A and ORX-B failed to inhibit TSH secretion in primary cultures from rat pituitary (Samson and Taylor, 2001). Curiously, plasma TH levels showed no changes after peripheral ORX-A administration (Mitsuma et al., 1999). It has been suggested that this lack of effect could be based by the slow metabolism of TH (Russell et al., 2000), which normally shows a delay in its secretory responses to modulatory factors (Kim et al., 2000).

Moreover, ORXs can modulate the activity of both the locus coeruleus (LC) and the basal forebrain neurons involved in the complex mechanism of arousal (Berridge et al., 2010; Tortorella et al., 2013), thus playing a relevant role in arousal during waking and suppressing rapid eye movement (REM) sleep (Hagan et al., 1999; Bourgin et al., 2000; España et al., 2001). As reported by Zitnik (2016) ORX-containing neurons from the hypothalamus innervate LC neurons that project to prefrontal cortex, implicating the involvement of LC in ORX-mediated EEG activation and wakefulness, with specific thalamic-cortical oscillatory rhythms (Del Cid-Pellitero and Garzón, 2011). On the other hand, ORX-mediated activation of the LC appears to be critical not only to maintain wakefulness during the active period, but also in the transition from sleep to waking. Such a transition is attributed to its engagement in neural circuit, contributing to the regulation of circadian rhythms that may exist through ORX-containing neurons in the DMH projecting to the LC, since it receives projections from the suprachiasmatic nucleus (SCN; Aston-Jones et al., 2001; Gompf and Aston-Jones, 2008).

Indeed, *in vitro* electrophysiological experiments showed that ORXs activate the tuberomammillary nuclei ablations (TMN; Bayer et al., 2001; Eriksson et al., 2001) and LC neurons (Hagan et al., 1999). Furthermore, *in vivo* studies demonstrated the involvement of the LC and OX1R in the LC neurons (Mochizuki et al., 2011) and OX2R signaling in the TMN, in ORX-induced arousal. Although conflicting, recent studies have shown that the ORX-mediated sleep-to-wake transition in mice is not dependent on the histaminergic system and mice deficient in both OX1R and hystamine 1 receptors display normal sleep/wakefulness patterns (Tsujino and Sakurai, 2009). Moreover, T4, entering the brain via the choroid plexus, is preferentially delivered to subependymal brain structures. High concentrations of LC norepinephrine promote active conversion of T4 to T3, leading to the preeminence of the LC as a site of T3 concentration (Dratman and Gordon, 1996).

Not secondarily, ORXs seem to play a positive role in the learning and memory functions, suggesting a sort of direct association with the regulation of the arousal system.

In fact, ORXs and their receptors are widely distributed throughout the brain and thereby regulate learning and memory functions (Jaeger et al., 2002; Telegdy and Adamik, 2002; Akbari et al., 2007). Specifically, ORX-A enables the acquisition, consolidation and retrieval of learning and memory (Jaeger et al., 2002; Yang et al., 2013). Studies show that ORX-A administered into the brain ventricles in conscious rats facilitates learning and the consolidation of learning, as well as retrieval processes in passive avoidance tests (Jaeger et al., 2002; Telegdy and Adamik, 2002; Ito et al., 2008). On the other hand, the electrical activity of hippocampal pyramidal neuron seems to be under direct control of the ORX system (Riahi et al., 2015), pinpointing the crucial role of hippocampal neurogenesis in learning and memory (Deng et al., 2009; Jessberger et al., 2009; Coras et al., 2010). Studies of behavior and hippocampal synaptic plasticity indicate that ERK_1/2_ activation enhances the induction of long-term potentiation, which contributes to the formation of memories, moreover the improvement in learning and memory mediated by ORX-A involves OX1R-mediated ERK_1/2_ activation and hippocampal neurogenesis (Selcher et al., 2003; Zhao et al., 2014).

**Figure 1:**
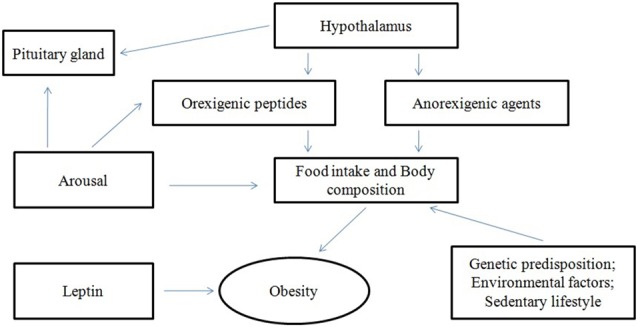
**Interactions of orexins (ORX)**.

It has been suggested that ORX-A is also implicated in the regulation of hormones, including prolactin (PRL; Hagan et al., 1999; Russell et al., 2000) and the luteinizing hormone (LH; Pu et al., 1998; Tamura et al., 1999), and in the control of the hypothalamo-pituitary-adrenal (HPA; Kuru et al., 2000; López et al., 2010), thyroid (Mitsuma et al., 1999; Kok et al., 2005) and somatotropic axes, as well (Hagan et al., 1999; Figure 1).

Conversely, the effect of the ORX system in cognition may be properly explained by its relationship with thyroid function along life span and not only by lowering TSH levels, frequently it related to cognitive impairment or elderly decline (Winkler et al., 2015; Chachamovitz et al., 2016; Moon, 2016). TH plays an essential role in normal brain development and its function linked to the maturation of its receptor in the developing brain. On the other hand, hypothalamus plays a crucial role in the regulation of TH serum concentrations, since the earliest stages of life (Alkemade, 2015), also causing specific cognitive alteration in progeny (Pasquali et al., 2015).

## Role of Orexins in Endocrine System

Despite their primary role as hypothalamic neuropeptides, ORXs and OXRs are widely expressed also in regions beyond the brain, in particular in endocrine tissues (Heinonen et al., 2008; Grimaldi et al., 2014). For example, the ORX peptide/receptor system was observed in the pituitary (Date et al., 2000; Blanco et al., 2001; Silveyra et al., 2007). The relevance of ORXs in the pituitary is also highlighted by the evidence that ORX-A-like molecule and OX1R are widely present in the adenohypophysis of *Xenopus* (Suzuki et al., 2007; Chieffi et al., 2014), and this underlines a highly conserved function. In the *Xenopus*, ORX-A-like substance is synthesized with and/or without TSH from TSH-containing cells and controls the functions of PRL-containing cells via OX1R in a paracrine fashion. In the Nile tilapia (*Oreochromis niloticus*, Monda et al., 2008; Suzuki et al., 2009), an ORX-B-like substance might be secreted from LH- or TSH-containing cells and might regulate the pituitary function, as well. Blanco et al. (2001) detected the presence of ORX-A and ORX-B in the human adenohypophysis cell types. The ORX-A was located in 33% of pituitary cells, including PRL (82 ± 5.3%), TSH (18 ± 2.3%), growth hormone (GH; 10 ± 2.3%), Follicle-Stimulating Hormone (FSH; 8 ± 2.6%) and LH (7 ± 3.2%) cells, but not in corticotrope cells (Monda et al., 2006, 2007).

*In vitro* studies showed that ORX-A stimulated PRL and GH secretion (Zieba et al., 2011), and that the intensity of this effect depended on the duration of the day. The PRL secretion stimulated by ORX-A was more intensive in the summer (long-day period), than in winter (short-day; Molik et al., 2008).

The ORX-B was observed in virtually all corticotrope cells of the adeno-hypophysis (Viggiano et al., 2010). According to this study, lactotrope cells are the major source of ORX-A and corticotrope cells of ORX-B, respectively, and these observations represent the cellular basis for a possible role of ORXs influencing virtually all the neuroendocrine axis, acting as signal molecules.

Though the adrenal gland seems to be a peripheral target organ for ORXs (Monda et al., 2004), additional studies revealed the presence of ORXs all over the neuroendocrine system, as neurotransmitter peptides in the regulation of GH, adrenocorticotropic hormone, thyroid, mineralocorticoid, and cortisol secretion (Pu et al., 1998; Molik et al., 2008; Figure 1).

Moreover, the relationship between thyroid gland metabolism and ORXs is supported by evidence in euthyroid subjects affected by narcolepsy without cataplexy (Sobol and Spector, 2014) and among canine models (Nishino et al., 1997; Riehl et al., 2000) of improvement in subjective sleepiness and mean sleep time in idiopathic hypersomnia due to levothyroxine administration. The proposed mechanisms include HPT axis alteration and/or activation of TSH, TRH, or T3 receptors in the brain’s arousal-promoting system (Shinno et al., 2011). On the other hand, low levels of circulating TSH are reported in ORX-deficient narcoleptic men, which could be attributed to low levels of plasma leptin and/or abnormal sleep-wake cycle (Kok et al., 2005). Finally, the prevalence of comorbid immunopathological diseases (also affecting thyroid gland) seems to be higher in subjects with narcolepsy and cataplexy with a significantly more severe prognosis (Martínez-Orozco et al., 2014).

## Orexins and the Thyroid Axis

The TRH is synthesized in the paraventricular nucleus (PVN; Lechan and Jackson, 1982), hosting a lot of neuroendocrine parvocellular neurons projecting to the median eminence, thus stimulating TSH secretion through endocrine and paracrine manners, respectively (Moura and Moura, 2004). Therefore, TSH synthesis and secretion are primarily regulated by TRH release and feedback inhibition by TH. However, PVN is not only the central point of the regulation of hypothalamic-pituitary-thyroid (HPT) axis, but it is also among the various brain areas to which orexinergic neurons project (Date et al., 1999). In fact, ORX neurons widely project to the autonomic part of the PVN (Peyron et al., 1998), in a strategic position to modulate the autonomic functions and the endocrine system. However, it is yet unknown whether either the ORX axons actually synapse with TRH neurons, or the ORX peptide/receptor system is involved in the regulation of HPT axis. The aim of this mini review is to investigate the relationship between orexigenic peptides and thyroid. The putative effects of ORX-A on HPT axis appear less clear because of the reported results in animal experiments investigating the effects of orexigenic peptides on the TSH release that appear inconclusive and conflicting. In some studies intracerebroventricular (ICV) injection of ORX-A in rats was shown to acutely decrease TRH release and plasma TSH concentration, with changes in plasma TH levels (Mitsuma et al., 1999). In particular, Mitsuma et al. (1999) observed that TSH suppression was due to *in vitro* TRH inhibition from the rat hypothalamus, following ORX-A addition, allowing the authors to conjecture that ORX-A was able to inhibit TRH release via the pituitary. Similarly, in another study Russell et al. (2002) observed that ICV administration of ORX-A significantly suppressed plasma TSH, without changes in plasma free T3 or T4 (FT3, FT4). The lack of effect on TH may be due to the low metabolism of TH. In this regard, it has been demonstrated that changes in FT4, occurred 15 h following iPVN injection (Kim et al., 2000). Besides it, another conflicting result was the lack of changes in plasma TSH, following twice-daily injections of ORX-A into the PVN for 3 days (Bartness et al., 2010). This result suggested the absence of a sustained effect of ORX-A chronically administered into the PVN on plasma TSH. Taken together, ORX-A displays no measurable effect on the HTP (Jones et al., 2001), whereas circulating TSH increased in response to central administration of ORX-B. However, Hagan et al. (1999) measured TSH solely at 40 min post-ICV administration, a time point to which TSH got normalized in Russell’s study (Russell et al., 2002), thus determining a confounding result. In another article, López et al. (2010) showed no significant change in hypothalamic prepro-ORX and OXRs mRNA levels in either severe hypo-, or hyper-hyperthyroid rats, in comparison to that in euthyroid ones, despite altered leptin levels. Since prepro-ORX-gene expression is regulated by leptin, this may suggest that PVN may not be an important anatomical site for chronic effect of ORX-A in the HPT axis. In a recent study on hyperthyroidism, Tohma et al. (2015) described the effects of hyperthyroidism on ORX-A, to investigate the putative relationship between ORX-A and the increased food intake, which characterizes hyperthyroidism. Although it seems that no link between ORX-A and increased appetite exists in hyperthyroidism, the ORX-A levels were decreased by hyperthyroidism, showing a negative correlation with FT3 and FT4 levels, and a positive correlation with TSH levels. The mechanism of these alterations is actually unknown. Probably, decreased ORX-A levels were the consequence of a compensatory mechanism for the increased basal metabolic rate, characterizing hyperthyroidism (Messina et al., 2015c; Moscatelli et al., 2015). Date et al. (2000) investigated the ORX-A levels in adult male rats affected by hyperthyroidism, observing no differences in ORX-A mRNA levels, caused by hyperthyroidism. Conversely, Kok et al. (2005) found changes in TSH concentration. Moreover, to investigate the impact of ORX-A upon the HPT axis, the circadian timing of its release was studied in a group of narcoleptic patients (Messina et al., 2015b; Triggiani et al., 2015; Valenzano et al., 2016).

In conclusion, this study showed that plasma TSH levels were reduced and were positively correlated to leptin plasma levels that could be the presumable cause of reduced TSH (Messina et al., 2015a). Moreover, among narcoleptic patients, energy imbalance, eating disorders (Chabas et al., 2007), precocious puberty, obesity and changes in bicarbonate levels are more frequent than in healthy subjects, also during childhood (Poli et al., 2013; Rocca et al., 2015; Franco et al., 2016).

Nevertheless, the relationship between leptin, sleep regulation and thyroid gland, as resumed in obese patients, affected by obstructive sleep apnea syndrome (OSAS) has to be considered. In fact, in these subjects, the nocturnal respiratory imbalance is a typical integrative part of the metabolic syndrome (Vgontzas et al., 2005; Basoglu et al., 2011). Moreover, there is an inverse link between sleep duration and body mass index, due to the increased levels of leptin and other systemic inflammatory markers during lifetime, which is independent of obesity in adults (Vgontzas et al., 2016). On the other hand, in non obese men and slightly obese women OSAS is similarly associated with HPA axis activation, albeit stronger, compared with obese individuals with sleep apnea (Kritikou et al., 2016), although with specific gender differences (Gaines et al., 2014). Again, acute sleep loss, in a less stressful environment, influences leptin levels in a way opposite to that of short-term sleep curtailment, associated with activation of the stress system. It is likely that sleep loss, associated with activation of the stress system, but not sleep loss *per se*, may lead to increased hunger and appetite and hormonal changes, which ultimately may lead to increased consumption of “comfort” food and thereby to obesity (Pejovic et al., 2010), because changes in energy homeostasis directly and reversibly impact the sleep/wake cycle (Collet et al., 2016).

About the role of thyroid ghrelin, leptin, and insulin levels did not differ accordingly to thyroid function conditions (Dubey et al., 2015; Kim et al., 2015), although leptin levels seem to correlate with thyroid autoantibody titers in non obese males (Maciver et al., 2015). More specifically, pediatric obesity is associated with higher TSH and lower FT4 concentrations and with a greater prevalence of abnormally high TSH and leptin concentrations that might in part explain obesity’s effects on thyroid status, perhaps through leptin’s influences on TSH secretion (Krause et al., 2016). In this framework, it has been well recognized that ORX neurons are regulated by peripheral metabolic cues, including ghrelin, leptin and glucose concentration, suggesting that they might provide a link between energy homeostasis and arousal states and the link between the limbic system and the ORX neurons might be important for increasing vigilance during emotional stimuli (Carotenuto et al., 2012, 2013; García-García et al., 2014). When imbalanced, such links could explain mood dysregulation and executive function impairment in obese OSAS and insomniac children and adolescents (Esposito et al., 2013, 2014a; Carotenuto et al., 2016).

## Conclusion

Although the knowledge of ORXs functions is evolving, studies of the putative effects of orexigenic peptides on the HPT axis remains conflicting and there are still a number of doubts concerning them. In fact, although the topography of ORXs and thyrotrope neural circuits suggests that TRH neuronal activity is influenced by ORX input, the nature of this input and the exact role of ORX (excitatory or inhibitory) remain unclear. However, it is clear that the ORX peptide/receptor system represents a novel molecular model system to understand the regulation of neuroendocrine system.

## Author Contributions

CDF, AM, VM, ME: conceived the study, participated in its design and wrote the manuscript. FM, AV, EV, SC, VDL: contributed to the conception and design. MC, GC, MM, GM: drafted the article and revised it critically for important intellectual content. GM: final approval of the version to be published. All authors read and approved the final manuscript.

## Funding

This study was supported by grants of Section of Human Physiology and Unit of Dietetic and Sport Medicine and Department of Experimental Medicine, Second University of Naples.

## Conflict of Interest Statement

The authors declare that the research was conducted in the absence of any commercial or financial relationships that could be construed as a potential conflict of interest.
